# Effect of Regorafenib on P2X7 Receptor Expression and Different Oncogenic Signaling Pathways in a Human Breast Cancer Cell Line: A Potential of New Insight of the Antitumor Effects of Regorafenib

**DOI:** 10.3390/cimb43030154

**Published:** 2021-12-09

**Authors:** Muhammed M. Salahuddin, Gamal A. Omran, Maged W. Helmy, Maha E. Houssen

**Affiliations:** 1 Department of Biochemistry, Faculty of Pharmacy, Damanhour University, Damanhour 22511, Egypt; msalahuddin@horus.edu.eg (M.M.S.); gamal.omran@pharm.dmu.edu.eg (G.A.O.); 2 Department of Biochemistry, Faculty of Pharmacy, Horus University, New Damietta 34518, Egypt; 3 Department of Pharmacology & Toxicology, Faculty of Pharmacy, Damanhour University, Damanhour 22511, Egypt; maged.helmy@pharm.dmu.edu.eg

**Keywords:** breast cancer, regorafenib, hypoxia, P2X7R, autophagy

## Abstract

Background: Breast cancer is the most common malignancy in women worldwide. P2X7 is a transmembrane receptor expressed in breast cancer and activated by the ATP tumor microenvironment, driving cell proliferation, angiogenesis, and metastasis via different signaling pathways. The role of the P2X7 receptor, hypoxia, and autophagy in regulating tumor progression is controversial. The multikinase inhibitor regorafenib prevents the activation of numerous kinases involved in angiogenesis, proliferation, and metastasis. The present study aimed to evaluate the modulatory effect of regorafenib on the hypoxia/angiogenesis/P2X7R/autophagy axis on the MCF7 breast cancer cell line and its impact on different signaling pathways involved in breast cancer pathogenesis. Methods: The levels of VEGF, VEGFR, PI3K, NF-κB, HIF-1α, and LC3-II were analyzed using ELISA, and caspase-3 activity was also assessed colorimetrically. Phosphorylated (p)-p38 MAPK and purinergic ligand-gated ion channel 7 (P2X7) receptor protein expression levels were analyzed via Western blotting. Reverse transcription-quantitative PCR was used to determine the mRNA expression levels of Beclin 1 (BECN1), LC3-II, and sequestosome 1 (p62). Results: Regorafenib reduced MCF7 cell viability in a dose-dependent manner. Furthermore, regorafenib significantly reduced levels of PI3K, NF-κB, VEGF, VEGFR, P2X7 receptor, and p-p38 MAPK protein expression, and markedly reduced p62 mRNA expression levels. However, regorafenib significantly increased caspase-3 activity, as well as BECN1 and LC3-II mRNA expression levels. Conclusions: Regorafenib was demonstrated to possibly exhibit antitumor activity on the breast cancer cell line via modulation of the P2X7/HIF-1α/VEGF, P2X7/P38, P2X7/ERK/NF-κB, and P2X7/beclin 1 pathways.

## 1. Introduction

Breast cancer is one of the most common cancers worldwide, and is the main leading cause of cancer mortality in women [1]. Angiogenesis, autophagy, and apoptosis serve essential roles in breast cancer progression via numerous signaling pathways [2,3,4].

Angiogenesis is a key contributor to the formation of tumors and metastases in a number of different malignancies [2]. VEGF is an angiogenic protein that is regulated by hypoxia-inducible factor-1 α (HIF-1α). HIF-1α is a transcription factor that regulates oxygen homeostasis, angiogenesis, and hypoxia response [5]. Hypoxia promotes tumor heterogeneity and plasticity, as well as the development of more invasive and resistant tumor subtypes [6]. VEGF stimulates the PI3K/AKT signaling pathway, protein kinase C, and the MAPK/ERK signaling pathway, which leads to the activation of endothelial cell proliferation, migration, and survival [7].

Apoptosis and autophagy are common types of programmed cell death, and their malfunction can contribute to tumor growth [8]. Autophagic cell death is mediated by the extensive degradation of organelles, which is dependent on autophagic flux [9]. During cancer treatment, crosstalk between the autophagy and apoptosis signaling pathways has been reported. Moreover, autophagy can induce apoptotic cell death [10,11]. Targeting apoptosis and autophagy via numerous signaling pathways, including the PI3K signaling pathway, has served a major role in cancer therapeutic development [12,13]

The purinergic ligand-gated ion channel 7 (P2X7) receptor is an ATP-gated nonselective cation channel receptor that serves a role in tumor growth regulation and progression, as well as in signal transduction during angiogenesis [14,15]. The P2X7 receptor is highly expressed in numerous types of cancer, including breast, prostate, and pancreatic cancer [15,16]. P2X7 receptor expression provides cancer cells with certain important properties, including improved engraftment strength and in vivo proliferation rate, higher expression of proliferation markers, decreased apoptosis, and accelerated VEGF production and angiogenesis [17]. In breast cancer, P2X7 receptor upregulation has been demonstrated to activate the AKT signaling pathway, the epithelial–mesenchymal transition, and govern the production of small extracellular vesicles that enhance invasion and migration [17].

Regorafenib is a Food and Drug Administration-approved multikinase inhibitor that inhibits various membrane-bound and intracellular kinases implicated in oncogenesis, angiogenesis, metastasis, and tumor immunity [18]. Therefore, the present study aimed to evaluate the modulatory effect of regorafenib on the P2X7/HIF-1α/VEGF, P2X7/P38, P2X7/ERK/NF-κB, and P2X7/beclin 1 pathways on the MCF7 breast cancer cell line and its impact on different signaling pathways involved in breast cancer progression.

## 2. Materials and Methods

### 2.1. Reagents

Regorafenib (BAY 73-4506; cat. no. S1178) was purchased from Selleck Chemicals. Trypsin (2.5%; 10X) was purchased from Gibco (Thermo Fisher Scientific, Inc.). FBS, DMEM, and MTT were purchased from Sigma-Aldrich (Merck KGaA). DMSO was purchased from SERVA Electrophoresis GmbH. Penicillin/streptomycin mixture and PBS were purchased from Lonza Group, Ltd. Ethanol was purchased from El Nasr Pharmaceutical Chemicals Co.

### 2.2. Regorafenib Solubility

The stock solution of regorafenib was dissolved in 1% DMSO and diluted in DMEM to obtain the concentrations (1.25, 2.5, 5, 10, 20, or 40 μM) used in the present study. DMEM with an equal volume of 1% DMSO as used for the treatment group was used as a control.

### 2.3. Experimental Cell Line

The human breast carcinoma MCF7 cell line was purchased from the American Type Culture Collection. MCF7 cells were grown in DMEM containing 1% penicillin/streptomycin and 10% FBS and incubated at 37 °C with humidified air and 5% CO_2_. The research protocol used in the present study was approved by the Ethics Committee of the Faculty of Pharmacy of Damanhour University (Damanhour, Egypt; approval no. 821PB22F).

### 2.4. Cell Viability Assay

The effect of regorafenib on cell viability was assessed using the MTT assay. The cells were cultured in DMEM containing 1% penicillin/streptomycin and 10% FBS in 96-well plates (4000 cells/well) at 37 °C overnight. The old media was then discarded, and 0.1 mL of DMEM containing regorafenib at concentrations of 1.25, 2.5, 5, 10, 20, or 40 μM was added to all wells, excluding the controls, and incubated for a further 72 h at 37 °C. Subsequently, cells were incubated for 4 h at 37 °C with 20 μL MTT reagent (5 mg/mL). The formed formazan crystals were dissolved in 150 μL of DMSO, and the absorbance was analyzed at 590 nm using a microplate reader (Bio-Rad Laboratories, Inc.). All experiments were carried out in triplicate. The viability of the cells was calculated as a percentage relative to that of the control groups. The median inhibitory concentration (IC_50_) values were calculated using CompuSyn software (version 1; CompuSyn, Inc.) [19].

### 2.5. Treatment of MCF7 Cells with Regorafenib and Experimental Design

Cells were grown in eight T-75 flasks (2 × 10^5^ cells/flask) overnight to allow for cell adhesion to the flasks in DMEM containing 1% penicillin/streptomycin and 10% FBS at 37 °C. The following day, flasks were divided into the following two groups and incubated at 37 °C for 72 h: (i) vehicle-treated group (1% DMSO); and (ii) regorafenib-treated group (IC_50_ level, 8.39 ± 0.43 μM).

### 2.6. Cell Lysate Preparation and Protein Quantification

RIPA lysis and extraction buffer (cat. no. 89900) was purchased from Thermo Fisher Scientific, Inc. and was used to acquire cell lysates. To MCF7 cell pellets, 1 mL of RIPA buffer was added and agitated gently on ice for 15 min. Cells were centrifuged at 14,000× *g* for 15 min, and subsequently, the supernatants were collected and stored at −20 °C. Total protein concentration was determined using the SMART^TM^ BCA protein assay kit (cat. no. 21071) purchased from Intron Biotechnology, Inc. The absorbance was analyzed at 562 nm.

### 2.7. Biochemical Analyses in Cell Lysates

The levels of VEGF and VEGFR2 were evaluated as markers of angiogenesis using ELISA. The Human VEGF ELISA Kit (cat. no. MBS355343; MyBioSource, Inc.) and the Human VEGFR-2/Flk-1 ELISA Kit (cat. no. CSB-E04763h; Cusabio Technology LLC) were used according to the manufacturers’ protocols. Moreover, PI3K and NF-κB levels were evaluated as angiogenic signaling mediators and markers of tumor growth and progression using ELISA. The Human PI3K ELISA Kit (cat. no. MBS268899; MyBioSource, Inc.) and the Human NF-κB p65 ELISA Kit (cat. no. MBS263235; MyBioSource, Inc.) were used according to the manufacturer’s protocols. LC3-II levels were evaluated as a marker of autophagy using ELISA. The Human Microtubule-Associated Protein 1 LC3B (MAP1LC3B) ELISA Kit (cat. no. MBS917498; MyBioSource, Inc.) was used according to the manufacturer’s protocol. The HIF-1α levels were evaluated as a marker of hypoxia using ELISA. The human HIF-1α ELISA Kit (cat. no. KA1247; Abnova Corporation) was used according to the manufacturer’s protocol. Caspase-3 activity was also assessed as a marker of apoptosis. The Human Caspase-3 assay colorimetric kit (product code CASP-3-C) purchased from Sigma-Aldrich (Merck KGaA) was used to determine caspase’3 activity according to the manufacturer’s protocol [20].

### 2.8. Reverse Transcription-Quantitative PCR (RT-qPCR)

The mRNA expression levels of LC3-II, Beclin 1 (BECN1), and sequestosome-1 (p62) were assessed as markers of autophagy. An RNA-spin ™ Total RNA Extraction Kit (cat. no. 17211; Intron Biotechnology, Inc.) was used to extract total RNA. The RNA was reverse-transcribed into complementary DNA (cDNA) using a HiSenScript ™RH (-) cDNA Synthesis Kit (cat. no. 25014; Intron Biotechnology, Inc.). The mRNA gene expression levels of p62, LC3-II, and BECN1genes were quantified using the TOPreal ™ qPCR 2X PreMIX (SYBR Green with low carboxyrhodamine dye, uracil N-glycosylase plus) Kit (Enzynomics Co., Ltd.). The primer pairs (Sigma-Aldrich, Merck KGaA) used for RT-qPCR can be seen in Table 1. Three experimental repeats were performed. β-actin and GAPDH were used as internal reference genes. Relative p62, LC3-II, and BECN1 mRNA gene expression levels were quantified using Applied Biosystems 7500 Real-Time PCR Software version 2.0.6. (Applied Biosystems; Thermo Fisher Scientific, Inc.). The real-time PCR instrument was programmed for p62 and BECN1 genes as follows: 95 °C for 10 min, followed by 40 cycles at 95 °C for 15 sec and 56 °C for 30 sec. It was programmed for LC3-II as follows: 95 °C for 10 min, followed by 50 cycles at 95 °C for 15 sec and 58 °C for 15 sec.

### 2.9. Western Blotting

Western blotting was used to evaluate the P2X7 receptor as a marker of angiogenesis, and phosphorylated (p)-p38 MAPK as an angiogenic signaling mediator and marker of tumor growth and progression. Equivalent protein samples (30 µg/lane) were loaded onto 12% SDS-PAGE gels and then transferred to polyvinylidene fluoride membranes. The membranes were blocked with 3% BSA in TBST buffer for 1 h at 37 °C. The primary antibodies were as follows: anti-P2X7R (1:1000; cat. no. DF8135) and anti-p-p38 MAPK (1:500; cat. no. AF4001); all from Affinity Biosciences, Ltd. The membrane was incubated with the primary antibodies for 2 h at 37 °C and was then treated with the secondary antibody, HRP-conjugated IgG (1:5000; cat. no. FNSA-0004, Wuhan Fine Biotech Co., Ltd.) at 37 °C for 1 h. Blots were evaluated using TMB solution (Cat.no. T0565; Sigma Aldrich, Merck KGaA). Image J software (National Institutes of Health) was used to analyze band densities normalized to β-actin protein expression levels, which were used as a loading control.

### 2.10. Statistical Analysis

Prism 5 for windows version 5.01 (GraphPad Software, Inc.) was used for data analysis. Data are expressed as the mean ± SEM. The Student’s unpaired t-test was used to compare statistical differences between groups; *p* < 0.05 was considered to indicate a statistically significant difference.

## 3. Results

### 3.1. Effect of Regorafenib on MCF7 Cell Viability

**Figure 1:**
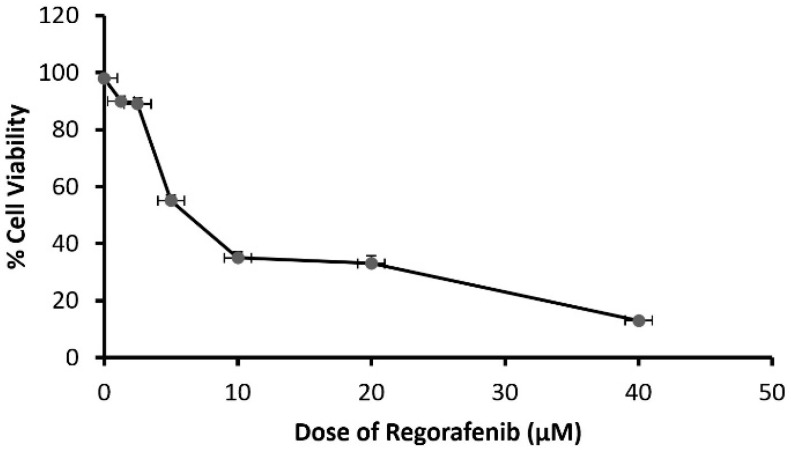
The viability of MCF-7 cells treated by regorafenib (1.25–40 μM).

Cells were exposed to a regorafenib concentration ranging between 1.25 and 40 μM. The results demonstrated that the cytotoxic effect of regorafenib on MCF7 cells was concentration-dependent, with an IC50 of 8.39 ± 0.43 μM (Figure 1).

### 3.2. Effect of Regorafenib on HIF-1α as Hypoxia Marker

**Figure 2:**
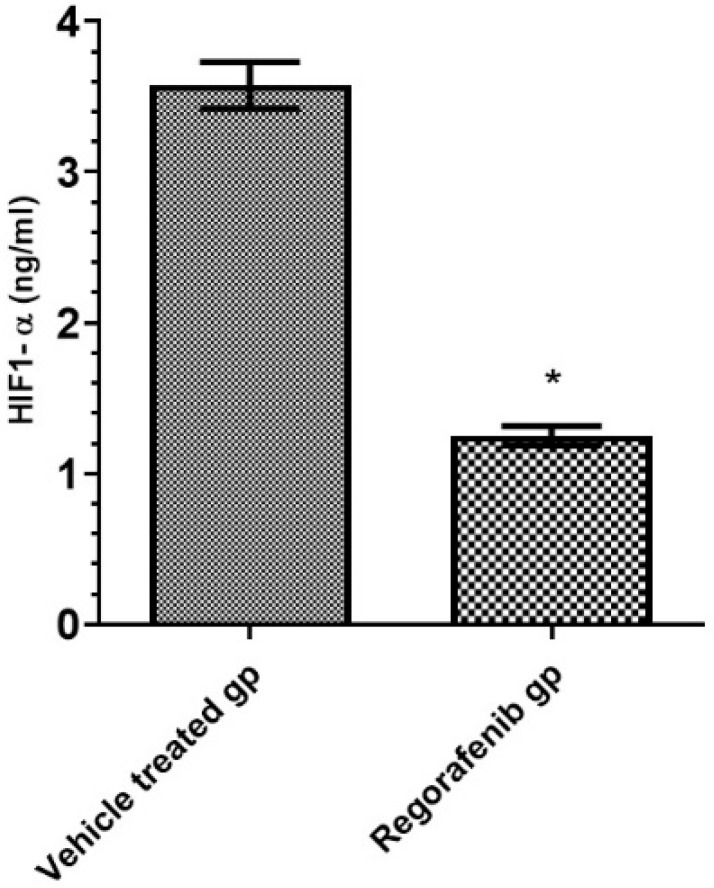
HIF-1α level (ng/mL) in regorafenib-treated and vehicle-treated groups. * Significant from control group at *p* < 0.05.

A significant decrease in HIF-1α level was detected in the regorafenib-treated group compared to the vehicle-treated group (Table 2) (Figure 2).

### 3.3. Effect of Regorafenib on Angiogenic Markers

**Figure 3:**
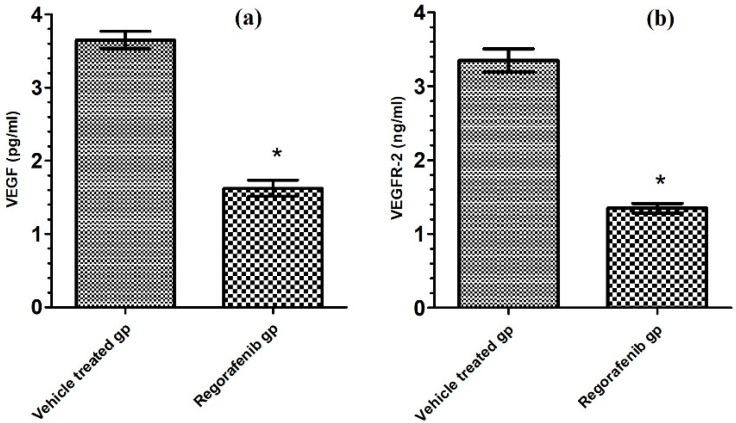
(**a**) VEGF level (pg/mL) in regorafenib-treated and vehicle-treated groups; (**b**) VEGFR-2 level (ng/mL) in regorafenib-treated and vehicle-treated groups. * Significant from control group at *p* < 0.05.

The regorafenib-treated group demonstrated a significant reduction in VEGF and VEGFR2 levels compared to the vehicle-treated group (Table 2; Figure 3).

### 3.4. Effect of Regorafenib on P2X7 Receptor Expression

**Figure 4:**
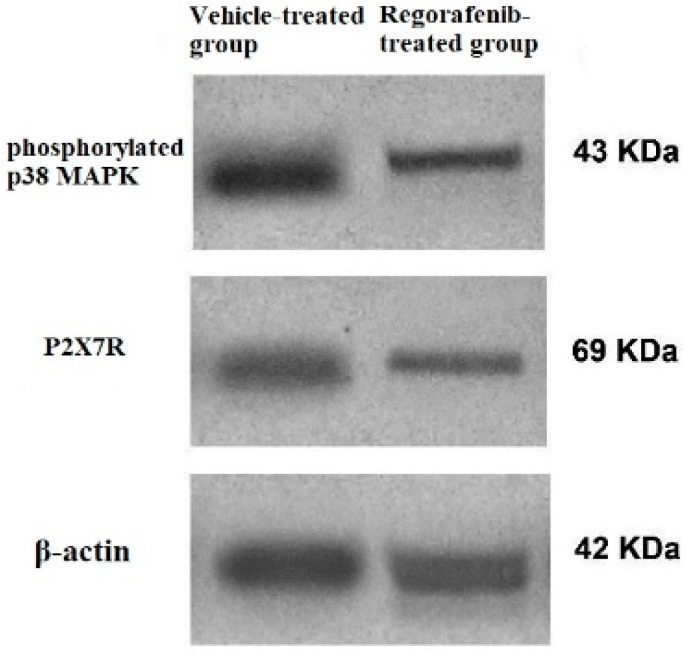
Western blot of P2X7R, phospho-p38 MAPK. and β-actin in regorafenib-treated and vehicle-treated groups.

Furthermore, the regorafenib-treated group demonstrated a significant reduction in P2X7R protein expression level by 62% compared to the vehicle-treated group (Figure 4).

### 3.5. Effect of Regorafenib on Markers of Tumor Growth and Progression

**Figure 5:**
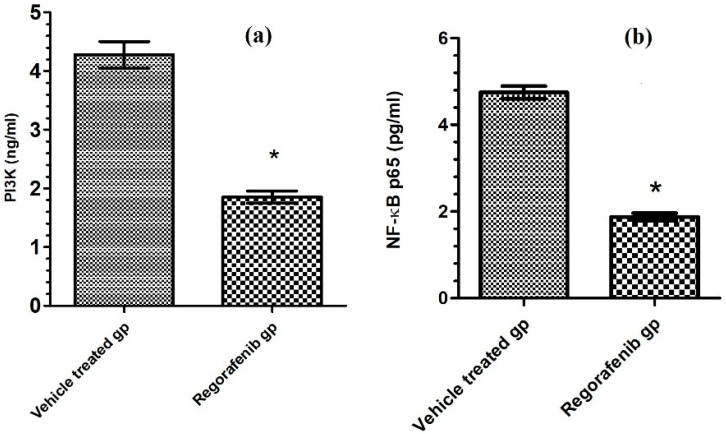
(**a**) PI3K level (ng/mL) in regorafenib-treated and vehicle-treated groups; (**b**) NF-κB p65 level (pg/mL) in regorafenib-treated and vehicle-treated groups. * Significant from control group at *p* < 0.05.

A significant decrease in the PI3K level was detected in the regorafenib-treated group compared to the vehicle-treated group (Table 2) (Figure 5). Furthermore, the regorafenib-treated group demonstrated a significant reduction in the NF-κB level and p-p38 MAPK protein expression level by 60.53 and 48%, respectively, compared to the vehicle-treated group (Table 2) (Figure 4 and Figure 5).

### 3.6. Effect of Regorafenib on LC3-II Level and LC3–II, p62, and BECN1 mRNA Expression Levels as Autophagic Markers

**Figure 6:**
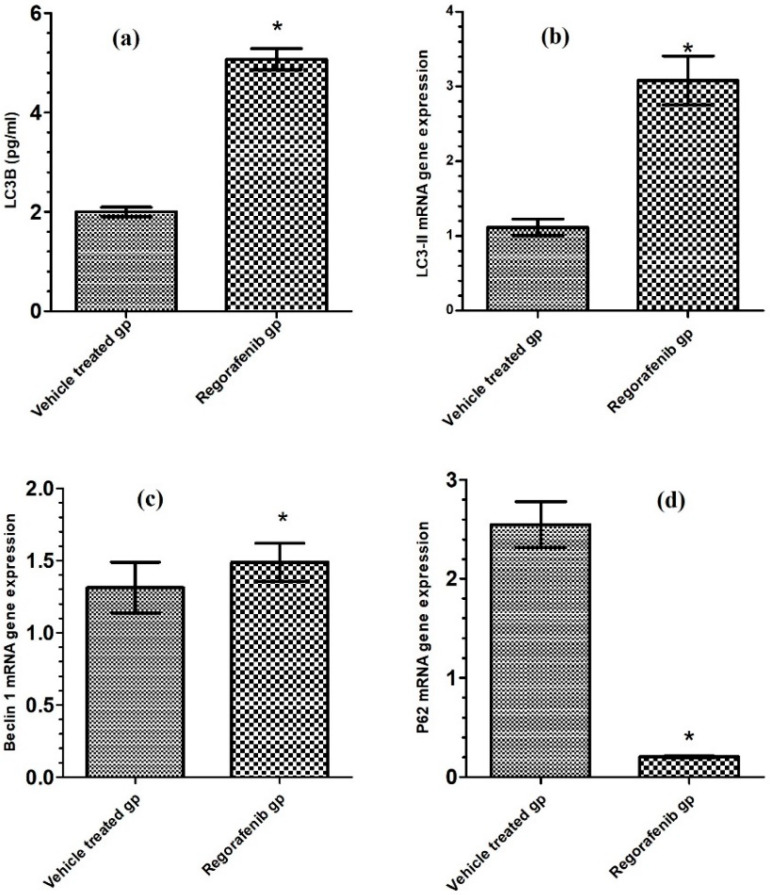
(**a**) LC3-II level (pg/mL) in regorafenib-treated and vehicle-treated groups; (**b**) LC3-II mRNA gene expression in regorafenib-treated and vehicle-treated groups; (**c**) BECN1 mRNA gene expression in regorafenib-treated and vehicle-treated groups; (**d**) P62 mRNA gene expression in regorafenib-treated and vehicle-treated groups. * Significant from control group at *p* < 0.05.

**Figure 7:**
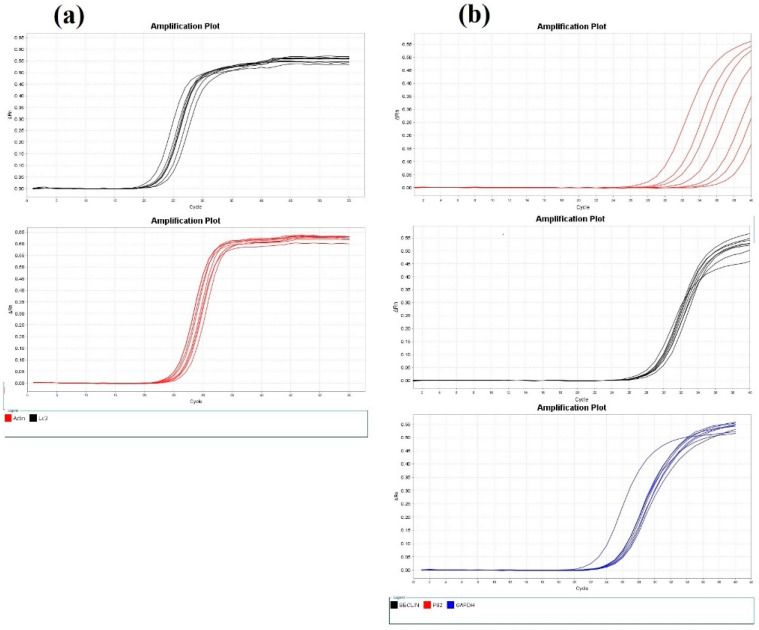
(**a**) Amplification plots of LC3-II and β-actin mRNA gene expression; (**b**) amplification plots of BECN1, p62, and GADPH mRNA gene expression.

The regorafenib-treated group demonstrated a significant increase in LC3-II level (Table 2), LC3-II mRNA gene expression levels, and BECN1 mRNA gene expression levels compared to the vehicle-treated group, by 153.75, 166.97, and 64.15% respectively. The p62 mRNA gene expression level was reduced in the regorafenib-treated group by 92.13% compared to the vehicle-treated group (Figure 6 and Figure 7).

### 3.7. Effect of Regorafenib on Caspase-3 as an Apoptotic Marker

**Figure 8:**
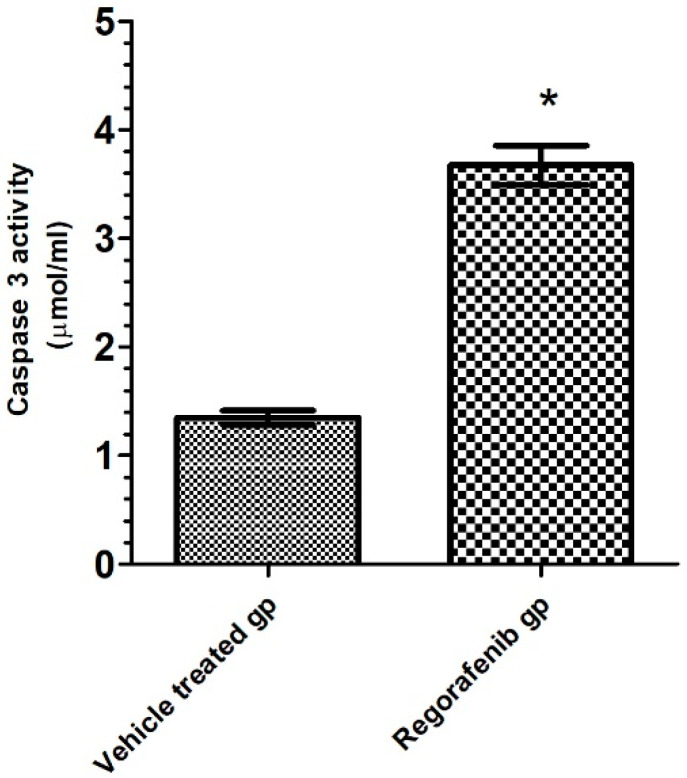
Caspase 3 activity (pg/mL) in regorafenib-treated and vehicle-treated groups. * Significant from control group at *p* < 0.05.

A significant increase in caspase-3 activity was detected in the regorafenib-treated group compared to the vehicle-treated group (Table 2) (Figure 8).

## 4. Discussion

Breast cancer is the most commonly diagnosed cancer, and the leading cause of cancer mortality in women worldwide [21]. Overexpression or dysregulation of receptor tyrosine kinases in breast cancer cells leads to accelerated tumor growth, angiogenesis, and metastasis by activating numerous downstream signaling pathways [22].

The P2X7 receptor is expressed in different types of cancer, and potentiates different oncogenic signaling mediating cancer progression [23]. Several calcium-related intracellular pathways involved in cell proliferation were shown to be activated by the P2X7 receptor. These include the JNK/MAPK, PI3K/AKT/myc, and HIF-1α/VEGF pathways [24].

Regorafenib successfully targeted numerous tyrosine kinases implicated in cancer, including those involved in oncogenesis, angiogenesis, and tumor microenvironment regulation [25]. Therefore, the present study aimed to evaluate the modulatory effect of regorafenib as a multikinase inhibitor on P2X7R expression and the P2X7/hypoxia/angiogenesis/autophagy signaling axis in the MCF7 breast cancer cell line.

To the best of our knowledge, this was the first study investigating the modulatory effect of regorafenib as a tyrosine kinase inhibitor on P2X7 signaling in breast cancer. The present study demonstrated that regorafenib reduced P2X7 receptor protein expression in MCF7 cells. Reduced expression of the P2X7 receptor was attributed to the reduction of HIF-1α expression, which is the main regulator of P2X7R expression [26]. This inhibition of P2X7R expression also resulted in inhibition of VEGF and PI3K/AKT expression levels, and ERK1/2 phosphorylation [26].

The present study also revealed that regorafenib may reduce angiogenesis by inhibiting the P2X7R/HIF-1α/VEGF axis, as mirrored by decreased VEGFR2 levels in MCF7 cells. This was in agreement with Mehta et al. [27], who reported that regorafenib significantly inhibited VEGF-A production in triple-negative breast cancer cell lines. This effect was attributed to regorafenib being a strong inhibitor of numerous tyrosine kinases, which are implicated in neovascularization and tumor progression via Raf inhibition [28].

Moreover, the present study also indicated that regorafenib may inhibit tumor growth and progression via the suppression of the P2X7/PI3K/mTOR/NF-κB axis and dephosphorylation of p-p38 MAPK in MCF7 cells. Similar results were reported in previous studies [29,30,31], which demonstrated that regorafenib diminished tumor progression via suppression of MAPK/ERK activation in hepatocellular carcinoma in vitro and in vivo. Furthermore, regorafenib has been reported to significantly inhibit NF-κB, p38, and ERK activation in bladder cancer in vitro and in vivo [30]. Another previous study determined that both the activation and expression of NF-κB-mediated proteins involved in tumor progression were suppressed by regorafenib treatment in colorectal cancer [29]. This suppression was attributed to VEGF and VEGFR2 production inhibition, which led to the inhibition of numerous intracellular signaling molecules, including PI3K/AKT kinase, phospholipase Cγ, protein kinase C, and MAPK/ERK signaling pathway proteins [7].

The present study also indicated that regorafenib may inhibit P2X7/HIF-1α/VEGF axis, as mirrored by reducing HIF-1α levels in MCF7 cells. HIF-1α is a major transcriptional regulator involved in the hypoxia response [32]. Multiple signaling cascades regulate HIF-1α expression at the transcriptional and translational levels. Inhibition of the P2X7R/PI3K/Akt signaling pathway has the potential to reduce HIF-1α expression [33]. NF-κB has been also shown to regulate HIF-1α activation. Inhibition of NF-κB can downregulate HIF-1α and VEGF [34,35]. The downstream of HIF-1α levels led to the blockage of the VEGF pathway, resulting in angiogenesis inhibition [36].

Furthermore, the present study indicated that regorafenib may activate autophagy, which was demonstrated by the increased BECN1 and LC3-II mRNA expression and decreased p62 mRNA expression. A previous study also reported that regorafenib induced autophagy in pulmonary fibroblasts as a result of the inhibition of the mTOR signaling pathway [31]. Such mTOR inhibition has previously been determined to reduce p62 expression contributing to autophagy induction [37]. Blocking the P2X7/beclin 1 signaling pathway in the MCF7 cell line downregulates the expression of the PI3K/AKT pathway which subsequentially activates autophagy.

In addition, the present study also suggested that regorafenib may induce apoptosis, which was demonstrated by increased caspase-3 activity in MCF7 cells. A recent study by Liu et al. [29] demonstrated a similar result, and reported that regorafenib triggered the intrinsic and extrinsic apoptotic pathways in colorectal cancer cells by activating caspase-3, -8 and -9 in vitro and in vivo. This result was attributed to the inhibition of the P2X7/PI3K/AKT/mTOR signaling pathway by regorafenib. Inhibition of this pathway has previously been demonstrated to directly block the phosphorylation of apoptosis signaling molecules (Bad, caspase-9, and acinus) or indirectly inhibit the activity of NF-κB and suppress the transcriptional activation of antiapoptotic genes [38].

## 5. Conclusions

Regorafenib was demonstrated to possibly exhibit antitumor activity on a breast cancer cell line via, at least in part, modulation of the P2X7/HIF-1α/VEGF, P2X7/P38, P2X7/ERK/NF-κB, and P2X7/beclin 1 signaling pathways. Further future studies are warranted to firstly validate other modulatory effects of regorafenib on different P2X7 receptor signaling pathways involved in breast cancer pathogenesis using different breast cancer cell line, and secondary to investigate this promising therapeutic insight in different human cancers, such as colon cancer.

## Figures and Tables

**Table 1:** Primers used for RT-qPCR.

	Forward Primer	Reverse Primer
P62	5′-GGG GAC TTG GTT GCC TTT T-3′	5′-CAG CCA TCG CAG ATC ACA TT-3′
LC3-II	5′-GAT GTC CGA CTT ATT CGA GAG C-3′	5′-TTG AGC TGT AAG CGC CTT CTA-3′
BECN1	5′-GGC TGA GAG ACT GGA TCA GG-3′	5′-CTG CGT CTG GGC ATA ACG-3′
GAPDH	5′-ACC ACA GTCCAT GCC ATC AC-3′	5′-TCC ACC ACCCTG TTC CTGTA-3′
β-actin	5′-CAC CAT TGG CAA TGA GCG GTT C-3′	5′-AGG TCT TTG CGG ATG TCC ACG T-3′

**Table 2:** The effect of regorafenib in MCF7 cell lysate.

Variables	Vehicle-Treated Group (N = 4), $\bar{X}$ ± SEM	Regorafenib-Treated Group (N = 4), $\bar{X}$ ± SEM
VEGF (pg/mL)	3.650 ± 0.1190	1.625 ± 0.1109 *
VEGFR-2 (ng/mL)	3.350 ± 0.1555	1.350 ± 0.0646 *
Caspase 3 activity (mmol/mL)	1.350 ± 0.06455	3.675 ± 0.1797 *
PI3K (ng/mL)	4.275 ± 0.2287	1.850 ± 0.1041 *
NF-κB (pg/mL)	4.750 ± 0.1443	1.875 ± 0.08539 *
LC3-II (pg/mL)	2.000 ± 0.09129	5.075 ± 0.2136 *
HIF-1α (ng/mL)	3.575 ± 0.1548	1.250 ± 0.06455 *

* Significant from control group at *p* < 0.05.

## Data Availability

Data are contained within the article.
